# Bioleaching of lithium from jadarite, spodumene, and lepidolite using *Acidiothiobacillus ferrooxidans*

**DOI:** 10.3389/fmicb.2024.1467408

**Published:** 2024-11-13

**Authors:** Rebecca D. Kirk, Laura Newsome, Carmen Falagan, Karen A. Hudson-Edwards

**Affiliations:** ^1^Environment and Sustainability Institute and Camborne School of Mines University of Exeter, Cornwall, United Kingdom; ^2^School of Environment and Life Sciences, University of Portsmouth, Portsmouth, United Kingdom

**Keywords:** bioleaching, lithium, critical minerals, metal recovery, iron bio-oxidation

## Abstract

Lithium (Li) is becoming increasingly important due to its use in clean technologies that are required for the transition to net zero. Although acidophilic bioleaching has been used to recover metals from a wide range of deposits, its potential to recover Li has not yet been fully explored. In this study, we used a model Fe(II)- and S-oxidising bacterium, *Acidiothiobacillus ferrooxidans* (At. Ferrooxidans), to extract Li from three different minerals and kinetic modelling to predict the dominant reaction pathways for Li release. Bioleaching of Li from the aluminosilicate minerals lepidolite (K(Li,Al)_3_(Al,Si,Rb)_4_O_10_(F,OH)_2_) and spodumene (LiAl(Si_2_O_6_)) was slow, with only up to 14% (approximately 12 mg/L) of Li released over 30 days. By contrast, *At. ferrooxidans* accelerated Li leaching from a Li-bearing borosilicate clay (jadarite, LiNaB_3_SiO_7_OH) by over 50% (over 120 mg/L) in 21 days of leaching, and consistently enhanced Li release throughout the experiment compared to the uninoculated control. Biofilm formation and flocculation of sediment occurred exclusively in the experiments with *At. ferrooxidans* and jadarite. Fe(II) present in the jadarite-bearing clay acted as an electron donor. Chemical leaching of Li from jadarite using H2SO4 was most effective, releasing approximately 75% (180 mg/L) of Li, but required more acid than bioleaching for pH control. Kinetic modelling was unable to replicate the data for jadarite bioleaching after primary abiotic leaching stages, suggesting additional processes beyond chemical leaching were responsible for the release of Li. A new crystalline phase, tentatively identified as boric acid, was observed to form after acid leaching of jadarite. Overall, the results demonstrate the potential for acidophilic bioleaching to recover Li from jadarite, with relevance for other Li-bearing deposits.

## Graphical abstract

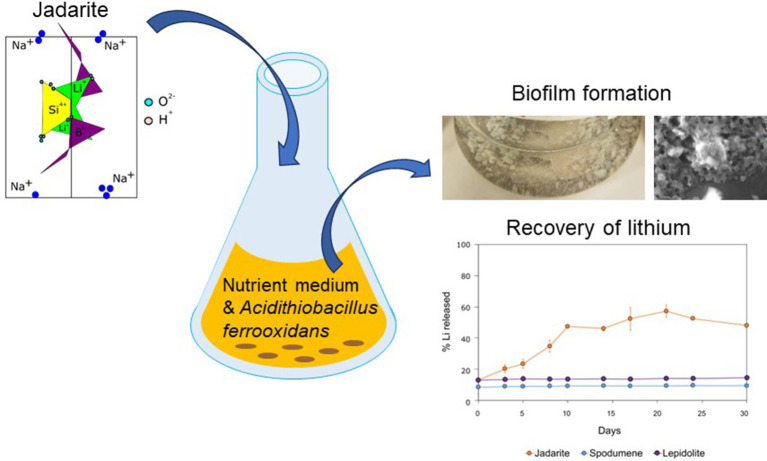

## 1 Introduction

Lithium (Li) is the lightest alkali metal in group one of the periodic table and has a wide range of applications from metallurgy and electrochemistry to medicine (Talens Peiró et al., 2013). It was added to the EU Critical Raw Materials list in 2017 (European Commission, 2017), and its demand increased by over 60% between 2019 and 2024 due to its use in technologies to aid in the energy transition to net zero, such as Li-ion batteries for vehicles (Greim et al., 2020; European Commission, 2017). The global maximum annual extraction demand for Li has been projected to increase to approximately 12 times the current demand by 2050 (Junne et al., 2020).

Li is found in a range of geological deposits, with closed-basin brines such as salars, estimated to represent 50–60% of the current estimated global resources. Pegmatites and Li enriched granites provide approximately 26%, Li clays 7% and Li-zeolites such as jadarite (LiNaB_3_SiO_7_) account for 3% (Jaskula, 2023; USGS Commodity Statistics, 2014). While Li concentrations in most terrestrial rocks are up to 60 mg/kg, high-concentration ore deposits such as pegmatites contain up to 8,000 mg/kg (Bradley et al., 2017; Cipollina et al., 2022) The highest concentrations of Li are seen in LCT-pegmatites (lithium, caesium, and tantalum); these deposits are a subset of granitic pegmatites containing spodumene (LiAl(Si_2_O_6_)) and/or lepidolite (K(Li,Al)_3_(Al,Si,Rb)_4_O_10_(F,OH)_2_) (Bradley et al., 2010). Jadarite may also become a significant source of Li; it is expected that from the Jadar deposit alone [Li-B deposit located in the Jadar Valley, Serbia (Stanley et al., 2007)], up to 1.6 million tons of Li could be extracted (Siljkovic et al., 2017). However, concerns with spatial planning, environmental impacts, and local politics are affecting project progression (Stefanović et al., 2023).

The main techniques used for Li recovery of spodumene and lepidolite are froth flotation and acid leaching. The flotation method involves preparing mineral pulps by adding deionised water adjusted to low pH with fatty acid collectors and flotation reagents such as CaCl_2_ that separate the minerals of interest (Xie et al., 2021), with Li_2_O yields of approximately 7.25% (Cerny et al., 1996). The acid leaching method uses high temperature, high-pressure roasting, and sulfuric acid (H_2_SO_4_) to recover Li from ores. This method can extract over 90% of the total Li from ores (Gao et al., 2020). These are energy-intensive processes with negative environmental consequences such as carbon emissions, water loss, ground destabilisation, ecosystem degradation, biodiversity loss, contaminated soil, and toxic waste. With the demand for Li increasing, the need to maximise each resource is paramount (Siezen and Wilson, 2009; Vera et al., 2022). Extraction techniques for materials such as jadarite are poorly documented in the literature, but low-temperature acid leaching methods may be suitable for Li release, depending on the chemical structure of the mineral.

Bioleaching involves using microorganisms to recover metals from minerals and rocks (Cerny et al., 1996). Chemolithoautotrophic organisms that can oxidise ferrous iron (Fe) or reduced inorganic sulphur compounds (RISCs), such as S°, S_2_O_3_^2−^, and S_4_O_6_^2−^, as electron donors (Siezen and Wilson, 2009) are used to generate acidity, which causes the release of metals within the mineral into solution or causes the metals to become fixed onto produced biomass or extracellular polymers in the system (Vera et al., 2022). These reactions are normally carried out at low pH (1–3) and have been highly successful in the bioleaching of sulphides for the extraction of copper (Cu) (Ristović et al., 2022). In materials containing low concentrations of reduced S and Fe(II), other microbes such as heterotrophs can be used for bioleaching. This involves the formation of organic acids and complexes that enhance metal release (Rezza et al., 2001); this process can be carried out over a wider pH range and can be manipulated for more alkali-rich materials such as battery wastes (Bahaloo-Horeh et al., 2018).

Leaching of Li from spodumene and lepidolite by fungi and heterotrophic bacteria has been investigated, with limited success. The results of these studies suggest that aluminosilicate structure may be a key control on the extent of leaching, and that biomechanical activity can play an important role (Rezza et al., 2001; Marcinčáková et al., 2015). Lepidolite bioleaching experiments caused the loss of muscovite, and fungal bioleaching led to the formation of a new silicate (quartz (SiO_2_)) phase identified through X-ray diffraction (Sedlakova-Kadukova et al., 2020; Liu et al., 2019). The biomechanical activity of microorganisms may be significant, as observed from fungal hyphae penetration of lepidolite colonised by *Aspergillus niger* (Rezza et al., 2001). Up to 95% of Li was recovered when bioleaching spent Li-ion batteries with *A. niger* (Horeh et al., 2016).

Acidophilic bioleaching by chemolithoautotrophs has previously been investigated for the recovery of Li from spodumene and lepidolite, as well as e-wastes and spent batteries (Roy et al., 2021). Up to 10% of the total Li was recovered when *At. ferrooxidans* was used to bioleach spent Li-ion batteries with data suggesting indirect bioleaching through acid generation as the predominant mechanism of metal release (Mishra et al., 2008; Moazzam et al., 2021). However, lepidolite bioleaching has been shown to be slow, and H_2_SO_4_ leaching has been proposed to be more effective (Liu et al., 2019). The first reported application of autotrophic bacteria (a mixed culture of mesophilic S-oxidising bacteria including high concentrations of *Acidiothiobacilli*) used in bioleaching was for zinnwaldite (KLiFeAl(AlSi_3_)O_10_(F,OH)_2_). In these experiments, 11 and 26% Li recovery was reported for batch and bioreactor experiments, respectively (Rezza et al., 2001). Bioleaching of Li from jadarite, mine waste, and mixed sediments is yet to be investigated.

A study comparing the bioleaching of Li by acidophiles and fungi reported up to 11 mg l^−1^ Li dissolved from lepidolite using a consortium of *At. ferrooxidans* and *Acidothiobacillus thiooxidans* equating to up to 8.8% recovery, with limited intracellular accumulation or extracellular fixation of Li observed. The *At. ferrooxidans* and *At. thiooxidans* consortium was more effective than *R. mucilaginosa* or *A. niger* for which only 1.1% of Li and 0.2% of Li were recovered, respectively. (Sedlakova-Kadukova et al., 2020; Roy et al., 2021).

The effectiveness of bioleaching by heterotrophic organisms, including bacteria and fungi such as *A. niger* is believed to depend on the presence of organic acids, which solubilise the minerals (Müller et al., 1995; Vandevivere et al., 1994). The results of spodumene leaching by *A. niger, R. rubra,* and *Penicillium purporogenum* showed adaptation of *R. rubra* and *P. purporogemum* to extreme, low nutrient environments that also contributed to Li release (Rezza et al., 1997).

The effects of deposit type and mineral structure on Li release through bioleaching are poorly understood, but identifying these may aid optimisation and choice of Li extraction method based on the chemical structure of the Li mineral. There are few data available on Li extraction from jadarite using conventional or bioleaching methods. In this study, a simple bioleaching mechanism containing *At. ferrooxidans* was used to investigate Li bioleaching from three deposit types via the oxidation of natural Fe presence within the minerals, compared to uninoculated controls. Through comparison with H_2_SO_4_ leaching, this study aims to outline the feasibility of bioleaching compared to conventional well-optimised leaching methods. Similarly, this study provides the initial data to understand Li leaching from jadarite through acid leaching and bioleaching methods. By adaptation of the shrinking core kinetic model, the results can be modelled to confirm the influence of bacteria in each reactive system compared to abiotic and acidic counterparts and therefore confirm whether the presence of *At. ferrooxidans* is necessary in leaching experiments. These data can be used to determine Li deposit types where exploration of bioleaching is worthwhile and to provide initial data on Li release from jadarite through both acid chemical leaching and bioleaching (Liddell, 2005).

## 2 Materials and methods

### 2.1 Minerals

The bioleaching of spodumene, lepidolite, and jadarite was investigated in this study. Samples of spodumene and lepidolite were collected from the Bikita mine (Zimbabwe) and donated by the British Geological Survey for the purpose of this study. Jadarite concentrate was collected from Jadar Valley (Serbia) and donated by the Natural History Museum, London. Spodumene makes up approximately 25% of the global Li resource, occurs in economically viable deposit grades (Swain, 2017), and is the most abundant and frequently mined Li-bearing mineral. Lepidolite also occurs in concentrations economically viable for extraction and hence has an established industry associated with it (Gao et al., 2023). The chemical structure of lepidolite is much more complex than that of spodumene (Lin et al., 2024). Jadarite is a less well-understood Li-bearing mineral that is not currently mined nor has an established extraction method. Jadarite has a very different chemical structure from spodumene and lepidolite, meaning that by comparing the three mineral types, the effect of chemical structure can be assessed. This provides a contrast between traditionally mined Li minerals and the novel jadarite borosilicate mineral to compare the effectiveness of bioleaching on these ore types.

Samples were acquired as dry consolidated rocks and sediments. They were characterised by grinding to approximately 0.5 μm using a Siebtechnik grinding mill, then analysed using X-ray diffraction (Siemens D5000 XRD) with the EVA identification software and database for spodumene and lepidolite, and against a reference jadarite spectra for the jadarite sediment. For bulk chemistry, 0.1 g of the samples were digested in an HF/HCl/HNO_3_/HClO_4_ mix (4-acid digest) at 180°C (Garbe-Schonberg, 1993), made up to 50 mL using Milli Q DI water [resistivity 18.2 MΩ·cm @ 25°C; total organic carbon (TOC) ≤ 5 ppb (Merck, 2023)] and analysed using an ICP-OES (Agilent 5110 VDV Inductively Coupled Plasma—Optical Emission Spectrometer). A ferrozine assay was used to estimate the proportion of bioavailable Fe(II) and Fe(III) in the materials using HCl-extractable Fe (Lovley and Phillips, 1986).

### 2.2 Microorganisms

The bacterium *At. ferrooxidans* was used in the experiments due to its documented success in Li bioleaching compared to other microorganisms (Sedlakova-Kadukova et al., 2020). This was obtained from an in-lab culture obtained from a mined ore (H2020-Nemo, n.d.). Cultures were maintained in a basal salt containing 7.5 g L^−1^ (NH_4_)_2_SO_4,_ 7.5 g L^−1^ Na_2_SO_4_.10H_2_O_,_ 2.5 g L^−1^ KCl, 25 g L^−1^ MgSO_4_, 2.5 g L^−1^ KH_2_PO_4_, and 0.7 g L^−1^ Ca(NO_3_)_2_ and a trace element solution (Ňancucheo et al., 2016) supplemented with 25 mM FeSO_4_ solution, adjusted to pH 1.8 using 5.5 M H_2_SO_4_. Cultures were grown in 50 mL of media at 28°C and 180 rpm. Cultures were maintained by subculturing in fresh media supplemented with 25 mM FeSO_4_ biweekly.

### 2.3 Bioleaching experiments and geochemical monitoring

Powdered mineral (2 g) was added to 190 mL of basal medium at pH 1.8 (adjusted with 5.5 M H_2_SO_4_) at 2% w/v concentration in sterile 250 mL conical flasks. These ‘bioleaching (biotic)’ experiments were inoculated with 10 mL of *At. ferrooxidans* (from stock grown for 14 days prior to inoculation), sealed with a foam stopper and foil cap to allow only airflow. These were left static to mimic heap leaching conditions at 23°C in a Thermo Scientific Heratherm incubator. All biotic experiments were conducted in triplicate. To quantify the role of microorganisms in metal leaching, ‘negative (uninoculated)’ controls consisting of 2 g of mineral were added to the basal medium without bacterial inoculation. To compare the results to a standard ‘chemical leaching’ system, 2 g of mineral was added to 8 mM H_2_SO_4_ (pH 1.8) solution. No Fe(II) was added to these to test the ability of *At. ferrooxidans* to oxidise Fe(II) present within the mineral.

All experiments were acidified to a final H_2_SO_4_ concentration of 8 mM equivalent to pH 1.8. For the bioleaching, uninoculated, and chemical leaching experiments 0.29mL of 5.5 M H_2_SO_4_ was added to produce this pH at a final volume of 200mL. Rewrite as Subsequent H_2_SO_4_ additions of 0.1–0.6mL were needed predominantly at day 0 and between day 13 to keep the pH at 1.8 in the jadarite bioleaching, uninoculated, and sulphuric acid experiments.

To monitor changes in geochemistry, 1 mL aliquots from each replicate were taken from the bioleaching experiments on days 0, 3, 5, 8, 10, 14, 17, 21, 24, and 30 and from the chemical leaching experiments on days 0, 2, 6, 8, 13, 16, 22, 27, and 30. The samples were then centrifuged at 10,000 *g* for 60 s, diluted with deionised water, acidified to 2% HNO_3,_ and stored at 3°C. To determine the amount of Li leached and to monitor the release of other metals from the minerals, concentrations of Al, B, Ca, Fe, K, Mg, Mn Li, S, P, Pb, and Zn in solution were measured using the ICP-OES (Aligent 5,110 series). pH was measured using a HANNA pH meter (calibrated with HANNA pH 1.68, 4.01, 7.01, and 10.01 standards). Following the bioleaching experiments, 1 mL aliquots were used to inoculate fresh media containing Fe(II) to test the viability of *At. ferrooxidans* by observing Fe(III) oxidation.

### 2.4 SEM imaging

A Thermo Fisher FEI Quanta 650F FEG-SEM was used to observe samples of jadarite taken before and after bioleaching to identify any structural changes and biofilm formation on the mineral surface. Samples were fixed with increasing concentrations of glutaraldehyde (0.75–2.5%) in phosphate-buffered saline solution and then dehydrated in increasing concentrations of ethanol (25–100%). The samples were subsequently deposited onto a silicon wafer, fixed to a pin stub with carbon tape (Newsome et al., 2018), coated using an Agar automatic carbon coater to approximately 25 nm in thickness, and analysed under high vacuum with secondary electron mode at 10.00 kV.

### 2.5 X-ray diffraction (XRD) analysis of bioleaching residues

After leaching, solid jadarite residues were washed in 15 mL MilliQ water to remove salts and allow drying, centrifuged at 2500 *g* for 10 min (Thermo Fisher Megafuge 40R), and left to dry for at least 24 h in a fume cabinet. These residues were powdered in an agate mortar and pestle and analysed using a powder XRD to determine changes in major mineral phases (Siemens D5000 XRD) and compared to similar spectra in the literature to find peaks not available in the EVA (Diffrac) database. Analysis of spodumene and lepidolite was not undertaken due to the lack of leaching observed based on no metal release or change to media composition through bioleaching.

### 2.6 Kinetic analysis

The shrinking core kinetic model was used to model the reaction kinetics for each system to predict whether acid leaching acted as a driver within the kinetic system (Fogler, 2020). The model was chosen based on the assumption that the conditions of the experiment were similar to that of acid leaching and previous successful use of the model to predict Li release from lepidolite in H_2_SO_4_ (Olaoluwa et al., 2023). This model describes experiments in which solid particles are consumed by reactions and are therefore described as ‘shrinking’. Reaction kinetics for heterogenous non-catalytically driven reactions can also be developed (Velardo et al., 2002). The shrinking core kinetic model has been used previously in predicting zinc oxide leaching from zinc-containing ore in high concentration H_2_SO_4_ and in lepidolite bioleaching using an *At. ferrooxidans* and *At. thiooxidans* consortium (Sedlakova-Kadukova et al., 2020).

The main assumptions for the model are as follows particles are spherical, shrinkage is uniform across the particles, the main reaction is the shrinking of the Li-containing minerals in the presence of the acidic media, other substances present in the ore (e.g., quartz, dolomite (MgCO_3_.CaCO_3_), and other non-Li bearing minerals) do not have any significant effect on the reaction or kinetics, the solids involved are non-porous and the reaction is dominated by outer diffusion, as in other lepidolite bioleaching experiments (Fogler, 2020; Didyk-Mucha et al., 2016). These assumptions are appropriate for the outlined experiments providing acid leaching is the predominant contributor to Li release.

The shrinking core model can be used to model chemical and diffusion-driven reactions, by using the relationship of these functions on reaction time (Equation 1).


(1) $$t=\frac{\rho_{Li-\mathrm{mineral}}}{{bM}_{Li-\mathrm{mineral}}c}\left\{\frac{r_{L}}{k_{0}}\left[{\left(1-1-x\right)}^{\frac{1}{3}}\right]+\frac{r_{L}^{2}}{6D_{c}}\left[1-\frac{2}{3}x-{\left(1-x\right)}^{\frac{2}{3}}\right]\right\}$$

where t is reaction time, *ρ* is the density of the mineral particles, b is the molar ratio of reactants, M is the relative molar mass of the Li mineral, c is the concentration of acid in media, r_L_ is the radius of the mineral particles, and D_C_ is the diffusion coefficient in the porous product layer.

Using the generalised equation for the shrinking core model, (Equation 2) the rate constant can be shown as k_r_ when assuming that the reaction is chemically controlled, and the reaction rate is much greater than the diffusion coefficient for the system:


(2) $$1-{\left(1-x\right)}^{\frac{1}{3}}=k_{r}t$$

where x is the Li leaching rate (determined by considering the amount of the total Li released by dividing Li released by total Li available from added mineral), k_r_ is the apparent rate constant and t is the leaching time (Equation 3).


(3) $$k_{r}=\frac{{kM}_{Li-\mathrm{mineral}}}{a\rho_{Li-\mathrm{mineral}}r_{0}}C_{A}$$

where k is the kinetic constant, M_Li-mineral_ is the relative molecular mass of the mineral, ρ_Li-mineral_ is the density of the mineral, a is a stoichiometric coefficient of reaction reagents, r_0_ is the initial radius of the mineral particle, and C_A_ is the concentration of acid.

## 3 Results and discussion

### 3.1 Material characterisation

The three samples contained Li-bearing phases and accessory minerals including quartz in the lepidolite and spodumene samples, and dolomite and probertite (NaCaB_5_O_7_(OH)_4._3(H_2_O)) in the jadarite sample (Table 1). The jadarite sample had the highest concentration of Li (2.2 wt.%), followed by spodumene (1.2 wt.%) and lepidolite (0.66 wt.%). Iron concentration was highest in jadarite (0.40 wt.%), followed by lepidolite (0.033 wt.%), and spodumene (<0.001 wt.%). The ferrozine assay indicated that bioavailable Fe(II) was present at concentrations of 0.020 mM in jadarite, 0.031 mM in lepidolite, and 0.023 mM in spodumene (Supplementary Table S2). Magnesium content was higher in jadarite (0.73 wt.%) when compared to lepidolite and spodumene in which Magnesium was <0.001 wt.% (Supplementary Table S1). The stoichiometry of Li, B, Fe, Ca, and Mg in jadarite could not be balanced solely by the presence of jadarite, dolomite, and probertite, suggesting the sample likely contained other poorly crystalline or amorphous minerals that were not identifiable using XRD.

**Table 1:** Minerals present in lepidolite, spodumene, and jadarite samples identified using XRD, with photographs of the samples as supplied and simplified 2D diagrams of the predicted Li-bearing mineral phases.

Ore type	Mineral	Formula(s)	Sample diagram	2D crystal structure
Lepidolite	Lepidolite Quartz	K(Li,Al)_3_(Si,Al)_4_O_10_(F,OH)_2_ Syn-SiO_2_	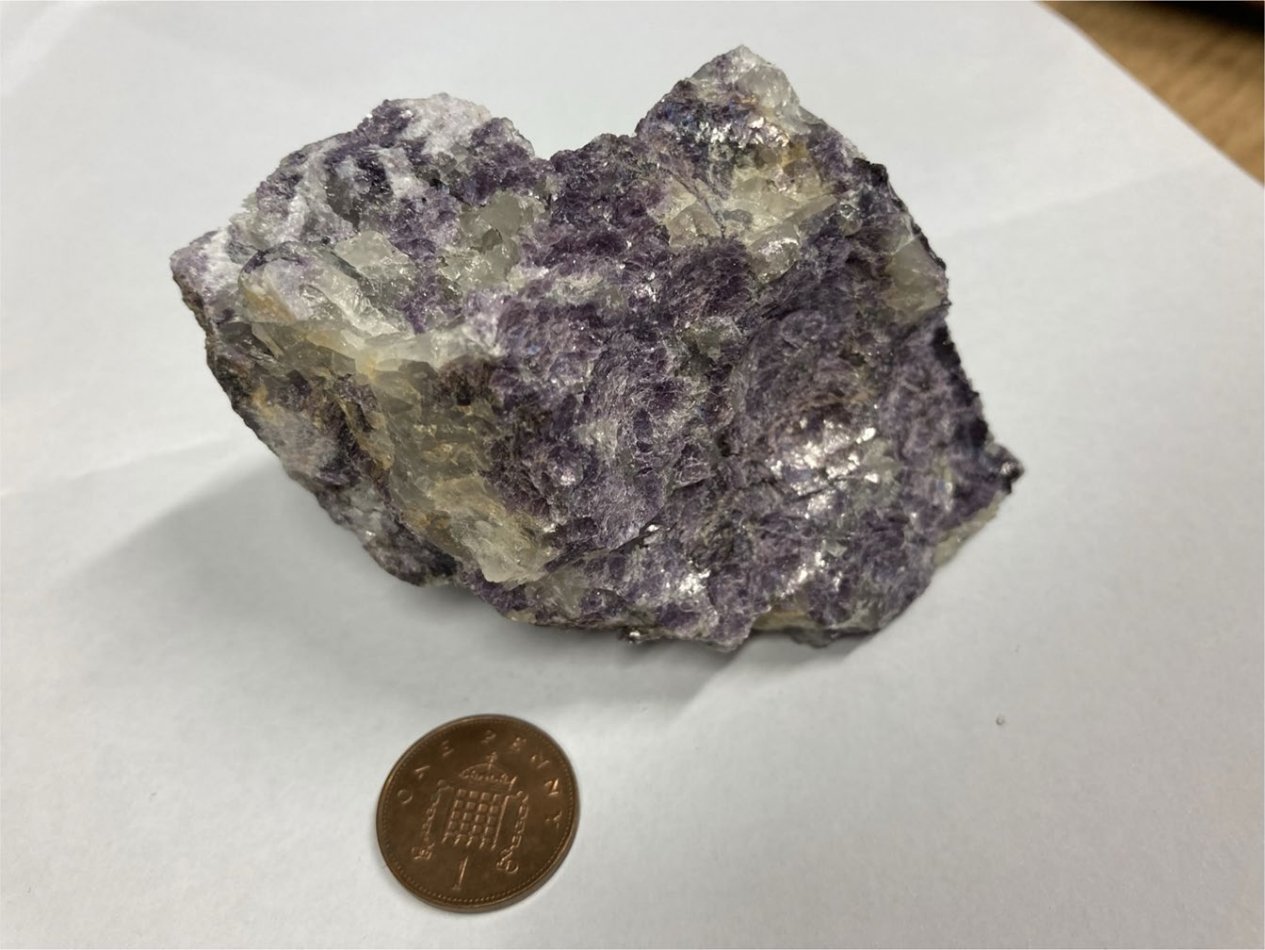	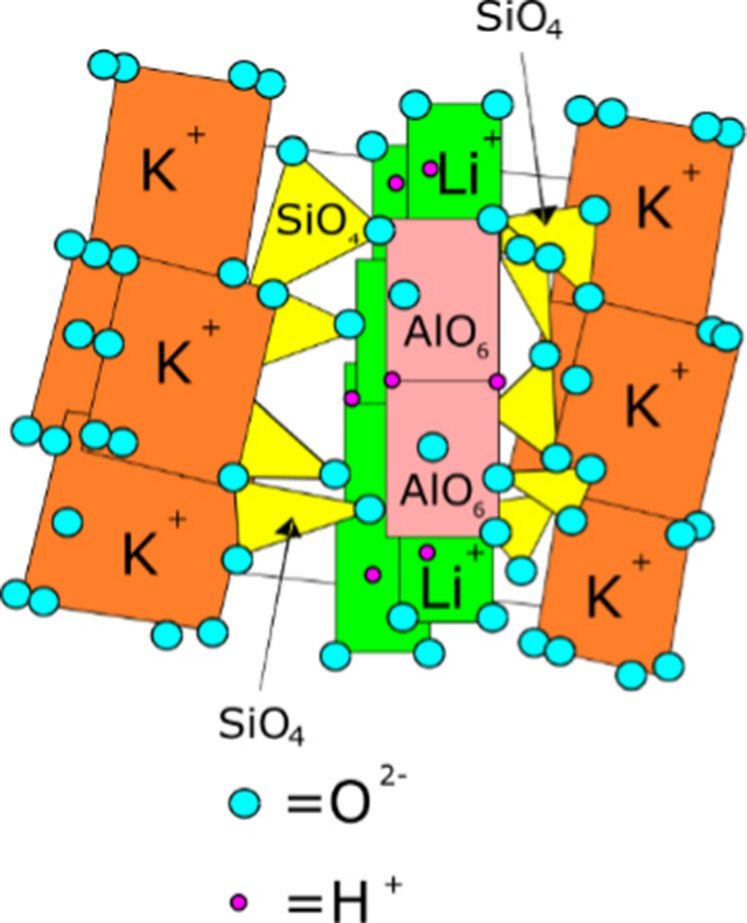

Spodumene	Spodumene Quartz	Alpha-LiAl(Si_2_O_6_) Syn-SiO_2_	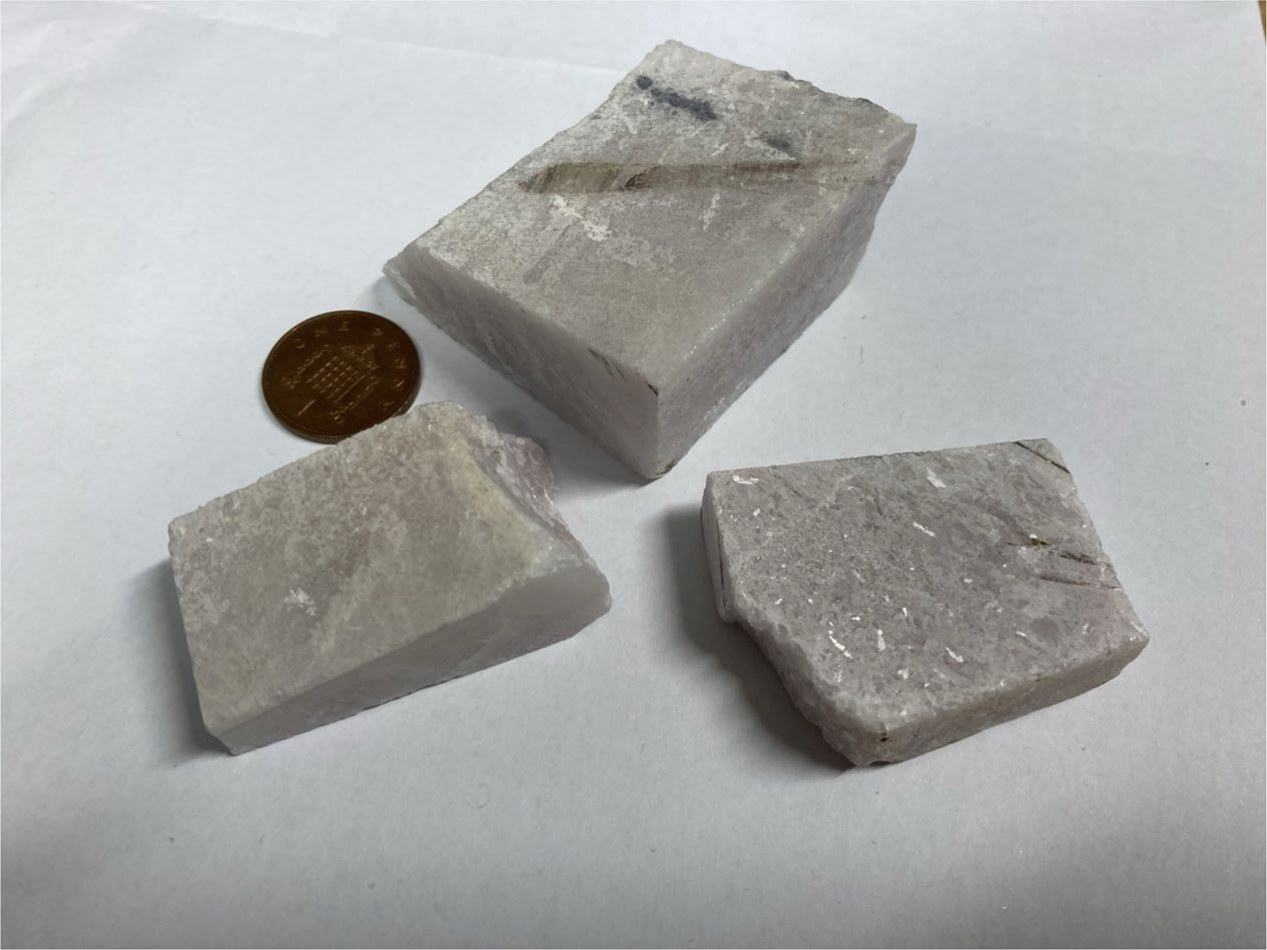	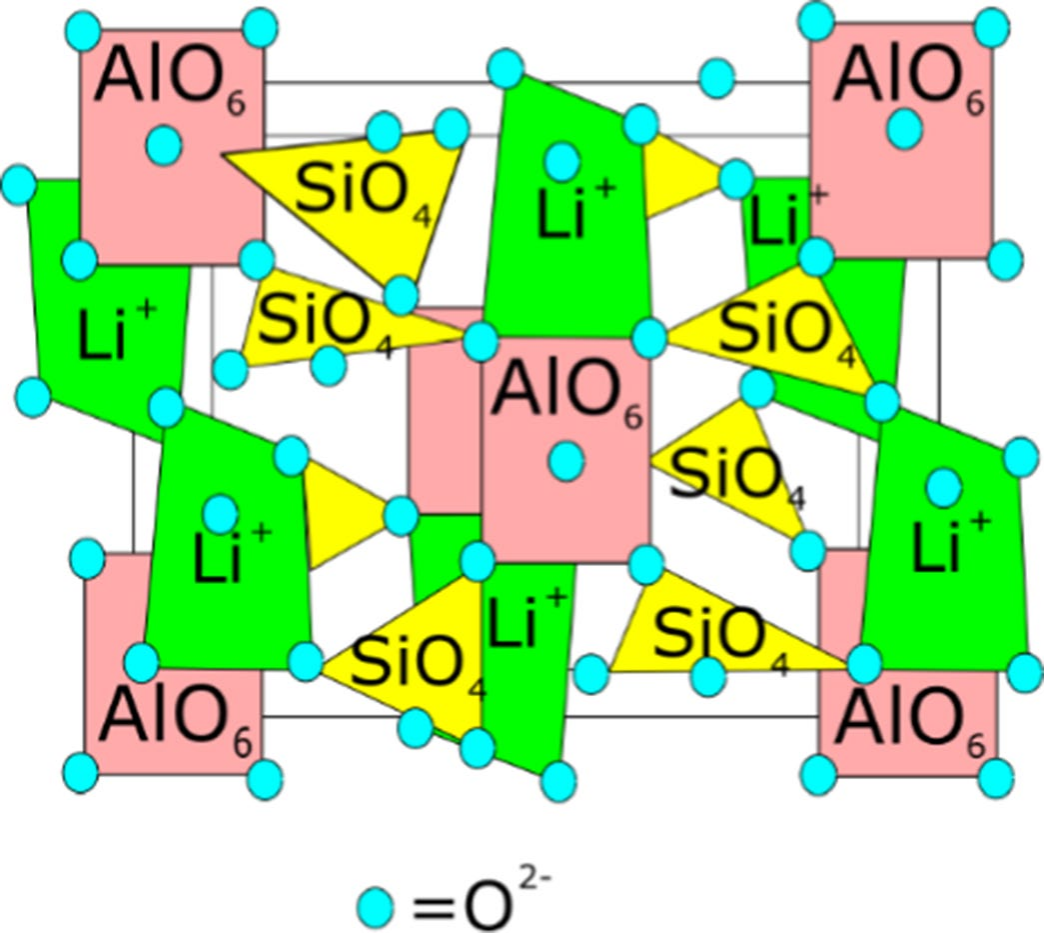
Jadarite	Jadarite Dolomite Probertite	LiNaB_3_SiO_7_ (Stanley et al., 2007) MgCO_3_.CaCO_3_ CaNa(B_5_O_7_(OH)_4_).3H_2_O (Gatta et al., 2022)	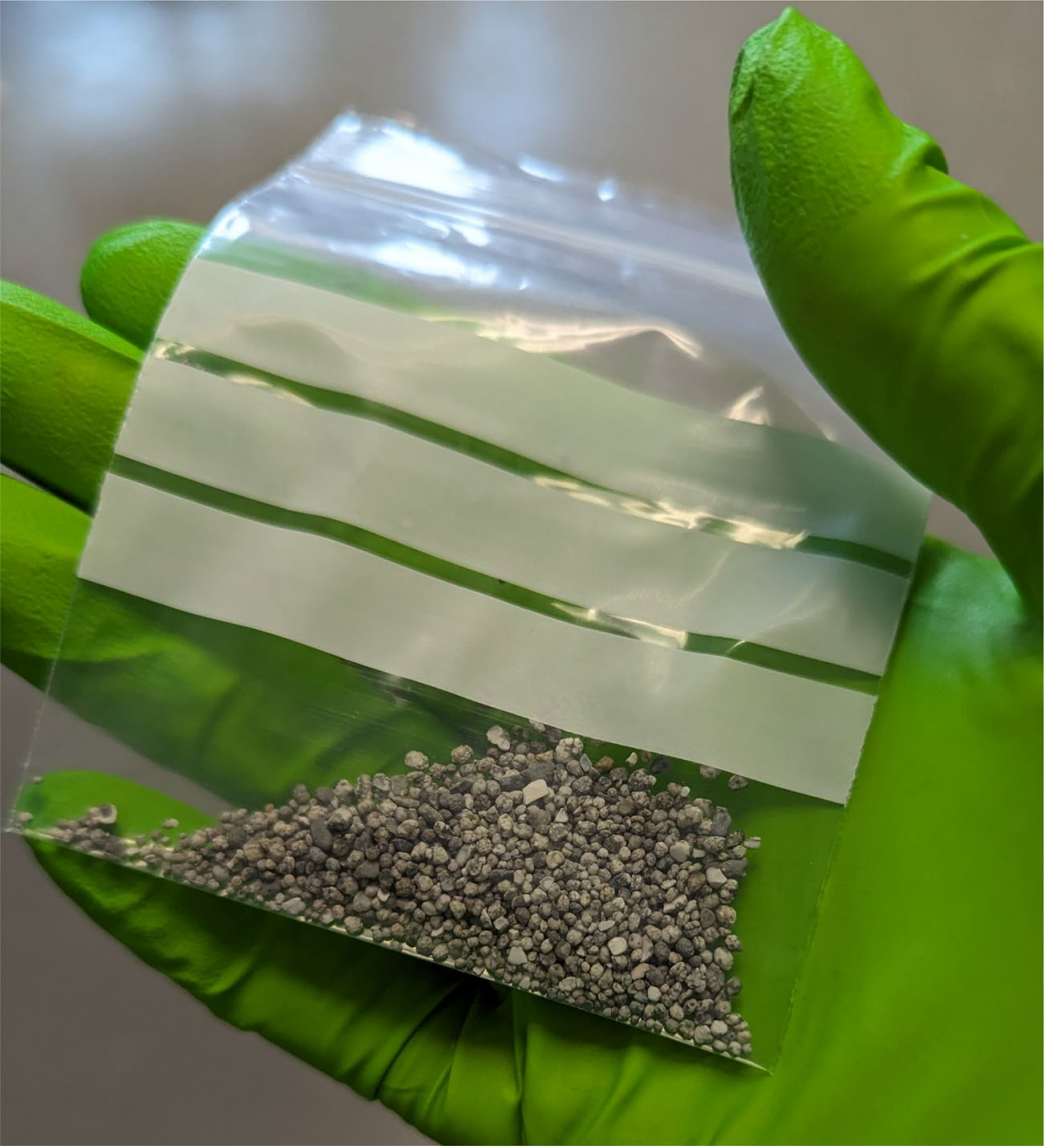	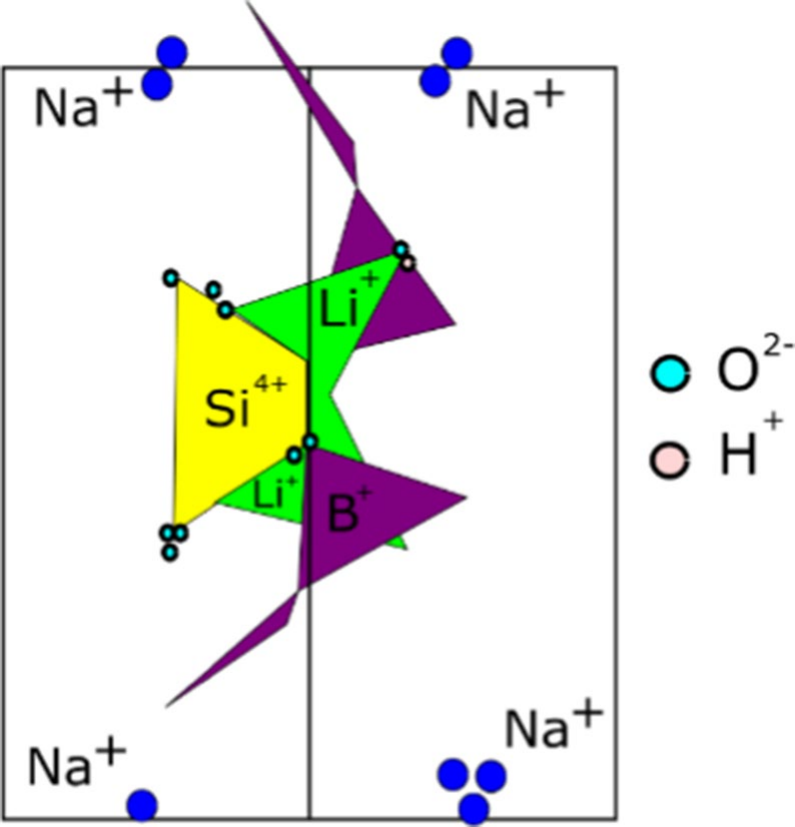

The crystal structures for lepidolite and spodumene were adapted from Su et al. (2019) and the single unit cell bonding structure for jadarite was adapted from Stanley et al. (2007).

### 3.2 Jadarite bioleaching

**Figure 1:**
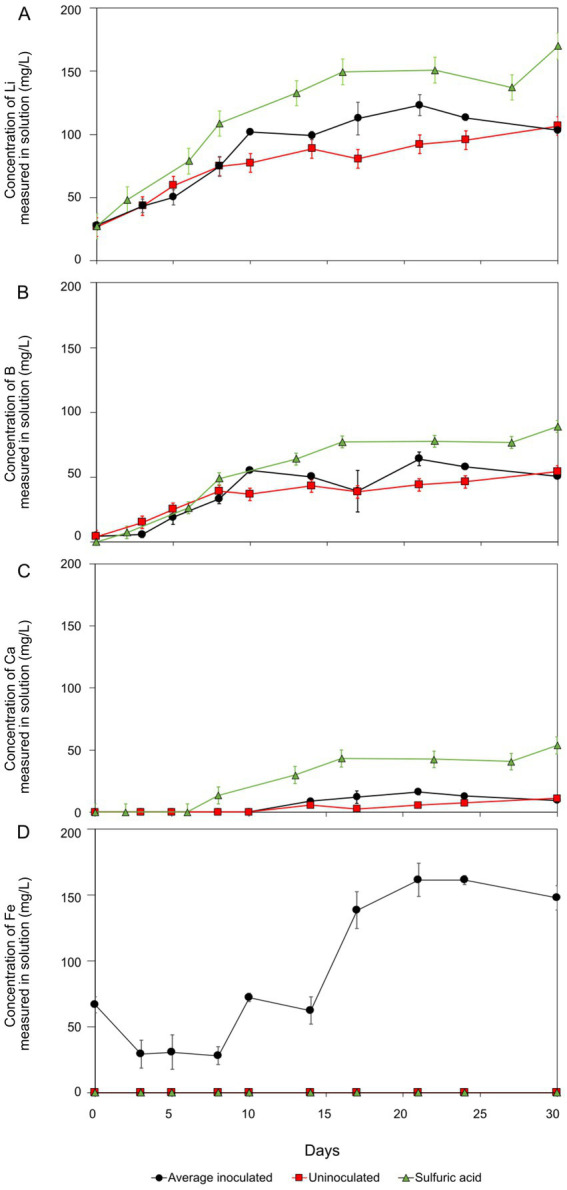
Lithium (A), boron (B), calcium (C), and iron (D) concentration during jadarite bioleaching (black circles), negative control (red squares), and acid leaching (green triangles). The results shown were obtained after subtracting the initial concentrations present in the basal medium. The error bars show standard error. Negative controls were not inoculated with *At. ferrooxidans,* and sulphuric acid leaching contained only 2,500 μL H_2_SO_4_.

The rate of jadarite bioleaching with *At. ferrooxidans* was highest in the initial stage of the reaction, from day 0 to day 10, after which the Li, B, and Ca concentrations plateaued (Figure 1A). A maximum concentration of 120 mg/L Li was recorded for the bioleaching experiments, representing approximately 57% of the Li within the mineral. This was approximately 24 mg/L higher than concentrations measured in the uninoculated control at this time point, demonstrating that *At. ferrooxidans* contributed to the leaching of Li from jadarite. The initial day 0 concentrations of approximately 30 mg/L Li in solution for all experiments may have been caused by surface Li release or release from exchangeable phases. By day 30, the Li concentration in the uninoculated negative control was similar to those in the biotic experiments, likely due to the decomposition of jadarite in the presence of H_2_SO_4._ Between days 10 to 25, the rate of Li release was more than 10% higher than in the uninoculated control. After this, the rate of Li release declined, possibly due to the loss of functionality of the bacteria due to the consumption of available Fe(II) from the mineral or to the Li being ‘armoured’ by secondary minerals or biofilms preventing surficial release. The H_2_SO_4_ chemical leaching released more Li than the inoculated and uninoculated acidic media leaches consistently throughout the experiment, with a final concentration measuring around 180 mg/L. The rate of Li release was comparable for both the bioleaching and acid leaching experiments. There are no available studies on Li release from jadarite by bioleaching, but the high concentration of Li released from H_2_SO_4_ leaching may indicate acid leaching contributing to some Li release from the inoculated bioleaching system. The 17 mM maximum Li concentration did not affect the functionality of the *At. ferrooxidans* once it had been inoculated into fresh media with 25 mM Fe(II) present for oxidation.

Boron (B) release was similar to that of Li for the biotic, uninoculated, and acid leaching experiments (Figure 1B). Approximately 64 mg/L B bioleached. Approximately 20 mg/L more B was released by bioleaching in the first 28 days than in the uninoculated control, suggesting the accelerated breakdown of Li–B bonds in jadarite in the bioleaching system. Boron release from the acid leaching was approximately 25 mg/L higher than the bioleaching system at the end of the 30-day experiment.

Controlling the pH was challenging within the jadarite system, possibly due to the presence of dolomite (MgCO_3_.CaCO_3_), which is known to buffer acidity (Roberts, 2016). Up to 20 mg/L in Ca and little change in Mg concentrations were documented (Figure 1C). The differences in the rates of Mg and Ca release suggest, that along with the dissolution of dolomite, other potentially non-crystalline Ca and Mg-bearing phases were also present and dissolved during the experiment. The final concentration of Ca in the acid-leaching system was more than double that of the bioleaching (53 mg/L) system and higher amounts of Ca were released in the bioleaching experiments compared to the uninoculated control experiments. The pH of the experiments increased from 1.8 to 7.0 immediately after the addition of jadarite, with the need for continuous adjustment of pH for the first 17 days to ensure the experiments remained in the pH 1–2 range required for bioleaching. The acid-leaching system required 31 and 18% more H_2_SO_4_ to maintain the pH range required for comparison with the bioleaching and uninoculated experiments, respectively (Supplementary Figure S1).

Soluble and therefore bioavailable Fe was present throughout the experiment (from the mineral) to support bacterial metabolism (Figure 1D), with a maximum of 47% of total Fe released in the biotic system and no release in the uninoculated control. The viability of the cells was confirmed at the end of the experiment by adding 1 mL aliquots to a basal medium containing FeSO_4_. Microbial Fe(II) oxidation was confirmed by the generation of Fe(III), which produced an orange colour in the medium.

**Figure 2:**
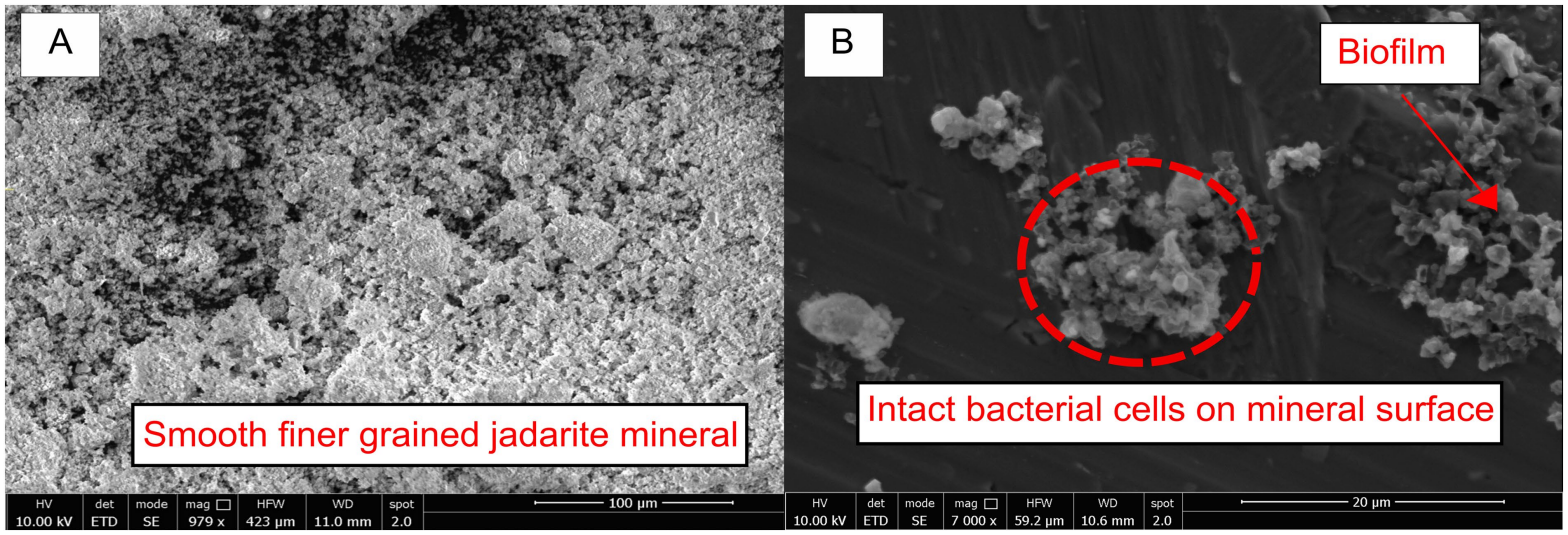
(A) Untreated ground jadarite sediment before exposure to the acidic basal medium and bacteria. (B) Jadarite sediment after bioleaching with flocculation, intact bacterial cells, and biofilm are indicated by the red arrow and label.

**Figure 3:**
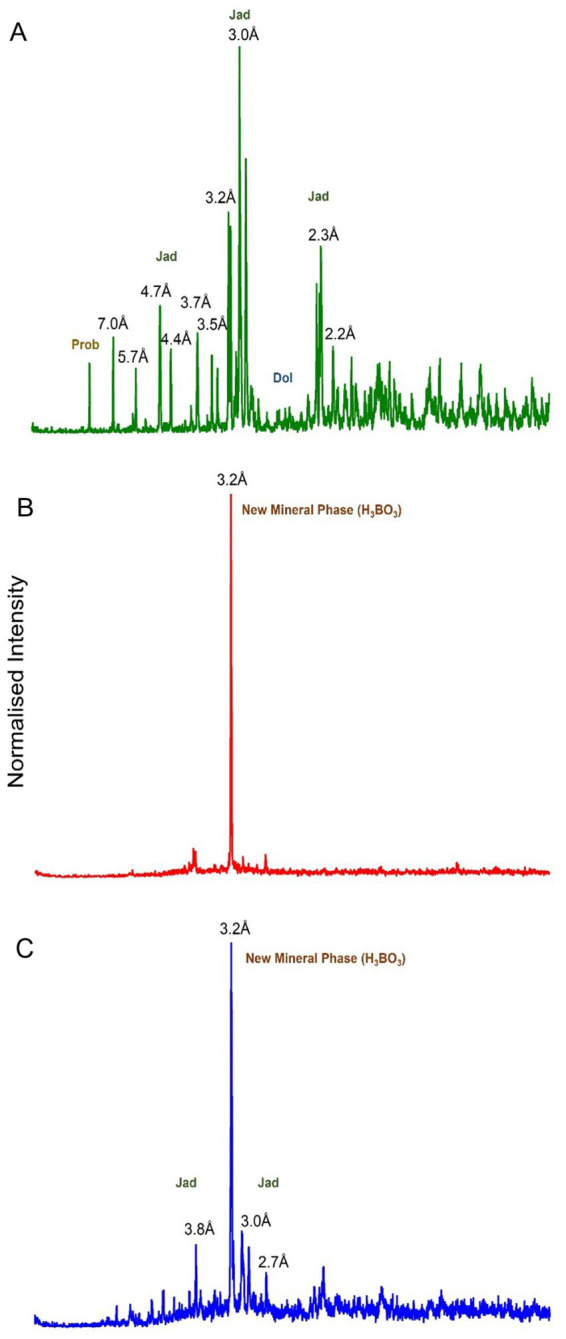
Powder XRD on a normalised intensity graph between 2θ = 0 ° and 2θ = 70 °. (A) Jadarite before leaching. (B) Jadarite post bioleaching. (C) Uninoculated cell control. Points labelled ‘jad’ indicate jadarite-related peaks, ‘dol’ indicates dolomite, ‘prob’ indicates probertite, and the new mineral phase was labelled.

Before bioleaching, the mineral surfaces were intact, and many showed regular crystal shapes (Figure 2A). After the experiments containing *At. ferrooxidans,* a shiny biofilm sheet was observed on the mineral surfaces, with some flocculation and clumping of the mineral also occurring (Figure 2B). Biofilm formation was identified by predominantly structural and morphological components observed through SEM analysis. The texture of the mineral surface in the biological systems varied greatly compared to that in the non-inoculated acidic system, and it had surface characteristics of bacterial biofilms seen in previous SEM studies of *Acidiothiobacillus* species (García-Meza et al., 2013). By contrast, no visible changes to the media or mineral were observed in the uninoculated control. Cells were present on many of the clumped mineral surfaces (Figure 2B), while some smoother surfaces appeared not to have cells present. The precipitation of new minerals through bioleaching was confirmed by XRD (Figure 3); therefore, variations in the mineral surface and biofilm presence may be due to the presence of the newly formed minerals. Since Li and B are undetectable by EDS spectra, characterising and observing variations in the minerals present after bioleaching is challenging.

The XRD analysis of samples from before and after bioleaching, as well as from the uninoculated control, revealed significant changes in the minerals present (Figure 3). The peaks for jadarite, dolomite, and probertite were no longer present in the bioleached sample, indicating their dissolution during the bioleaching experiment. The amount of Li dissolved, however, stoichiometrically exceeded that provided by the B in jadarite, suggesting that other unidentified poorly crystalline Li minerals were likely present in the sample. At the end of the experiment, a single new peak was present at 2θ = 27.9°, suggesting formation of a new solid phase. The same phase formed in the uninoculated control, which also retained some of the jadarite peaks with lower intensity, while no significant dolomite or probertite peaks were identified. The new solid phase material had a single peak that was fine and symmetrical indicating the presence of a highly crystalline phase (Jian and Hejing, 2003). The 2θ = 27.9° value may be indicative of boric acid (H_3_BO_3_) crystals precipitating from a boron-saturated solution in this acidic system (Figure 3B; XRD) (Sheikh et al., 2017; Ata et al., 2000). Boric acid has a triclinic structure when formed at 23°C, with 4 symmetric units of B(OH)_3_ providing the highly symmetrical crystalline structure for the sharp XRD peak (Alavia et al., 2023). Therefore, the reported XRD spectra directly indicated H_3_BO_3_ formation in the outlined conditions and are comparable to previously reported XRD spectra of crystalline H_3_BO_3_ (Mergen et al., 2004). Boric acid is categorised as a human reproductive toxicant, therefore the formation of a solid that contains boric acid would have serious implications for the industrial application of acid (bio) leaching of jadarite and warrants further investigation.

Overall, the experiments showed that jadarite was amenable to acid leaching and that the presence of *At. ferrooxidans* increased the rate of leaching and resulted in the formation of a biofilm that may have contributed to the dissolution of the mineral surface (Han et al., 2024). However, substantial quantities of acid were required to maintain the pH levels required for the bioleaching, uninoculated acidic media leaching, and H_2_SO_4_ leaching reaction to occur, likely due to the presence of dolomite, which may influence the potential of bioleaching to be applied on an industrial scale.

### 3.3 Spodumene bioleaching

**Figure 4:**
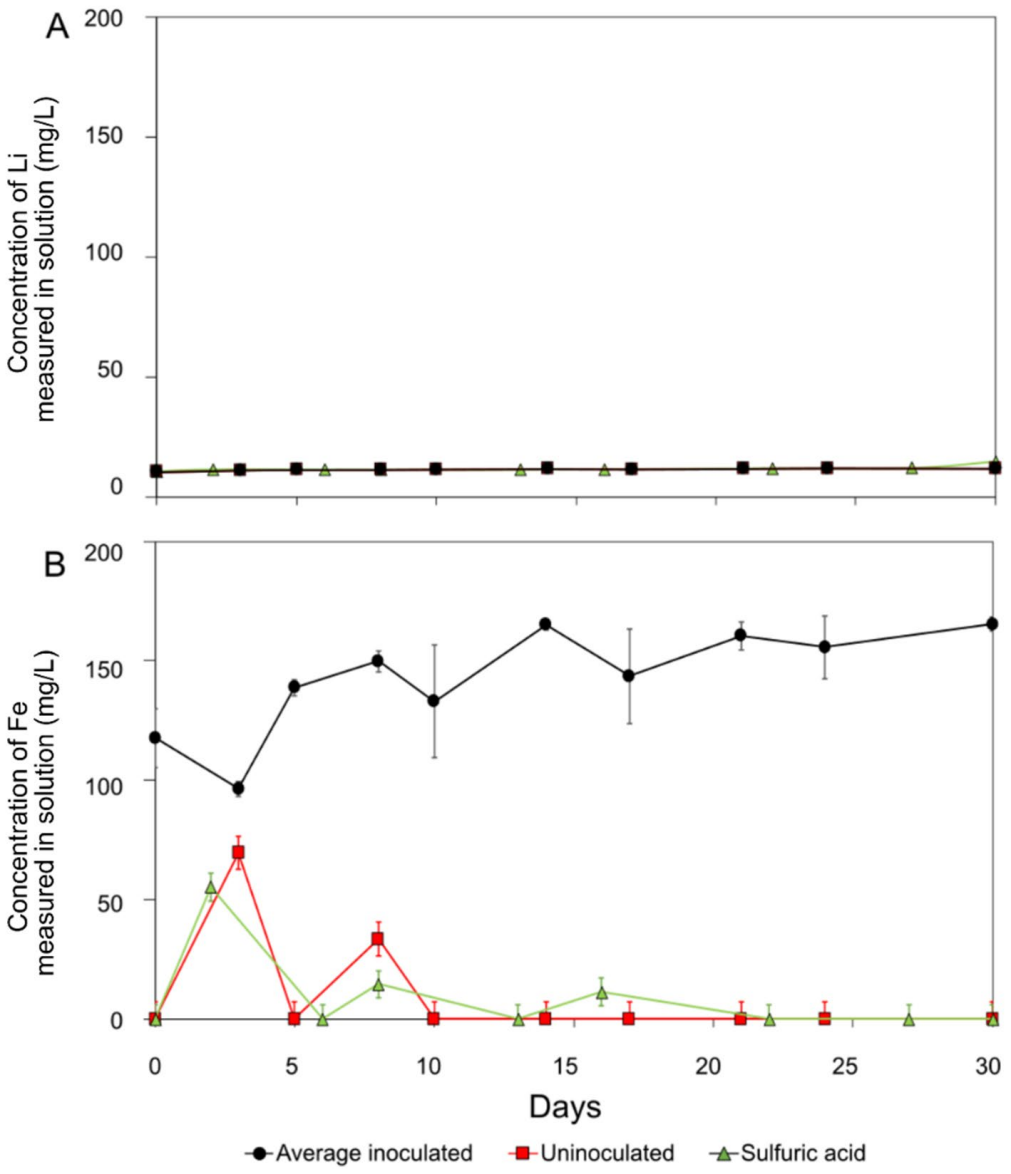
Spodumene bioleaching (black circles), negative control (red squares), and acid leaching (green triangles) results (A): lithium. (B): iron. The error bars show standard error. Negative (acidic) controls were not inoculated with *At. ferrooxidans*. Negative controls were not inoculated with *At. ferrooxidans,* and the sulphuric acid leaching contained only 2,500 μL H_2_SO_4_.

After 30 days, 12 mg/L of Li was released into the solution from spodumene, equating to approximately 9% of the total Li content of the mineral (Figure 4A). The pH remained constant at 1.8 for the whole experiment with no requirement for H_2_SO_4_ addition. Minimal changes to the appearance of the mineral were noted. The uninoculated negative control behaved similarly to those containing *At. ferrooxidans* throughout the experiment, with a maximum of 11 mg/L of Li measured in the solution. Only a small proportion of the Li in spodumene was susceptible to acid leaching, and bacteria did not enhance the rate or extent of leaching most likely due to Fe(II) oxidation not influencing the release of Li into the system due to the chemical structure of the mineral.

More Fe was solubilised from spodumene in the biotic system compared to the controls (uninoculated and acid leaching), demonstrating microbial cycling of Fe(II)/Fe(III) in this system (Figure 4B). The fluctuations in the negative control may have been due to heterogeneity in the system, for example, the release of exchangeable phases and precipitation. The initial concentrations in the biotic system (44% of total Fe present in solution) were much higher than the uninoculated control, indicating some carryover of Fe from the growth medium (Figure 4B).

### 3.4 Lepidolite bioleaching

**Figure 5:**
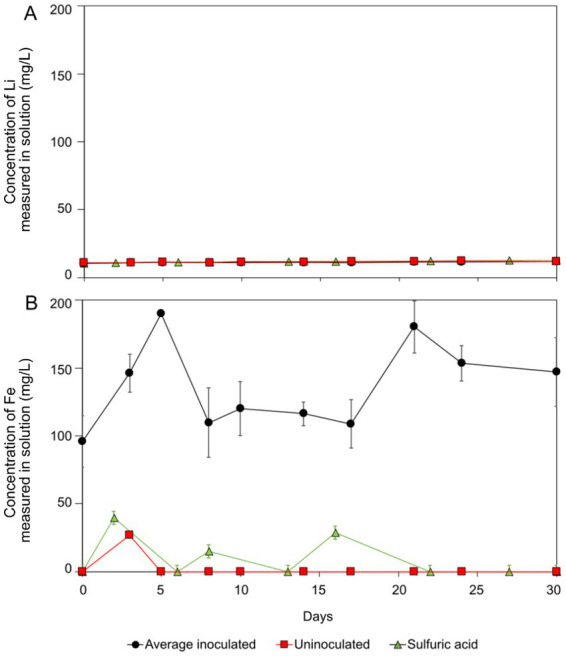
Lepidolite bioleaching (black circles), negative control (red squares), and acid leaching (green triangles) results (A): lithium. (B): iron. Error bars showing standard error. Negative controls were not inoculated with *At. ferrooxidans* and sulphuric acid leaching contained 2,500 μL H_2_SO_4_ only.

Lithium release from lepidolite was similar to that from spodumene, with the maximum Li concentrations in solution measuring at approximately 12 mg/L, equating to approximately 14% of the total Li concentration. This was similar to the negative control which yielded 12 mg/L Li over 30 days (Figure 5A). The pH remained constant at 1.8 with no H_2_SO_4_ additions, and there were no visible changes in the appearance of the mineral or solution throughout the experiment. In a previous study, a bacterial consortium yielded approximately 8% Li recovery from lepidolite (Horeh et al., 2016). Comparable with spodumene, the similarity of the experiments with *At. ferrooxidans* and compared to the uninoculated control suggests the bacteria had minimal impact on the leaching of Li, with abiotic acid leaching responsible for the small proportion of Li leaching that occurred.

Fe concentrations were elevated in the biotic experiments because of release from the mineral. The maximum concentration seen in the biotic system was 180 mg/L Fe, compared to no release in the uninoculated control (Figure 5B). Fluctuation in the aqueous concentration measurements implies changes in the proportion of aqueous and precipitated Fe by Fe(II) bio-oxidation by *At. ferrooxidans* during the reaction time. The trend was not observed in the uninoculated control (see Figure 5).

### 3.5 Comparison of the bioleaching experiments

**Figure 6:**
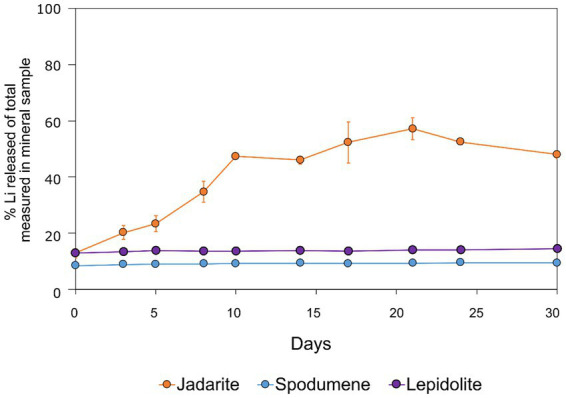
Lithium extraction yields (%) during bioleaching of jadarite (orange circles), spodumene (blue circles), and lepidolite (purple circles) from averages of triplicate experiments. The experiments were kept static at 23°C for over 30 days. The error bars represent averages across triplicate experiments.

The bioleaching and acid leaching of jadarite resulted in much higher concentrations and proportions of Li released into the solution than for of spodumene and lepidolite (Figure 6). The yield of Li release from jadarite was considerably higher in the presence of bacteria, whereas the role of the bacteria in the spodumene and lepidolite bioleaching experiments was insignificant due to their similarity to the uninoculated and acid controls (Jian and Hejing, 2003; Sheikh et al., 2017).

Iron release to the solution was enhanced in the biotic reactions for all ore types, indicating biomechanisms involved in solubilising Fe from Fe(II)-bearing minerals. *At. ferrooxidans* can oxidise Fe(II) to Fe(III) (Equation 4) or oxidise RISCs (Equation 5). However, it was difficult to determine if the bacterium was using only Fe(II) or both Fe(II) and RISCs as electron donors (Zhan et al., 2019; Harahuc et al., 2000). The Fe content of jadarite exceeded that of spodumene and lepidolite (Supplementary Table S1). Similar amounts of bioavailable Fe (approximately 0.02 mM of 0.5 N HCl-extractable Fe) were present in each of the three ore types. However, the changes in Fe concentrations observed in these experiments allude to the contribution of the Fe(II) bio-oxidation mechanism. Following Fe(II) oxidation, the formed Fe(III) can act as an oxidant that leaches the mineral and can be reduced back to Fe(II) for further oxidation by *At. ferrooxidans* (Equation 6) (Brock and Gustafson, 1976). The S concentrations were low in all samples (Supplementary Table S1) and no elemental S was added to the system, further indicating Fe oxidation mechanisms to be the main contributor to metal release. This cycling can further enhance the release of Li from the jadarite, as well as affect the concentration of solubilised Fe over time. Acidity was not produced during these experiments, with the pH remaining around 1.8 (spodumene and lepidolite) and increasing likely due to dolomite consumption (jadarite). This may suggest that *At. ferrooxidans* was predominantly metabolising via Fe(II) oxidation and Fe(II)/Fe(III) cycling.


(4) $$2{Fe}^{2+}+\frac{1}{2}O_{2}+2H^{+}\to 2{Fe}^{3+}+H_{2}O$$

(5) $$S^{0}+1\frac{1}{2}O_{2}+H_{2}O\to H_{2}{\mathrm{SO}}_{4}$$

(6) $${Fe}^{2+}⇌{Fe}^{3+}$$

The enhanced leaching of Li from jadarite is likely to be due to the mineral structure and bonding of Li, which is different from Li in spodumene and lepidolite. The Li in jadarite is bound in a tetrahedral borosilicate structure with Na^+^ ions situated between the layers (Table 1). Li appears in ‘triangular’-like bonding to B^3+^ and Si^4+^ in a lattice with O2-ions within the Li–B–Si bond space and Na^+^ in the free space (Table 1) (Whitfield et al., 2007). In contrast, it is theorised that the Li in spodumene is present within a tetrahedral 4-fold or 6-fold tetrahedral structure bound to O within the aluminosilicate structure, where Al forms octahedral bonds in the bonding chain. The cavities in the polyhedral host Li act as a high-energy bonding environment, which is difficult to overcome in low-energy reaction conditions (Li and Peacor, 1968; Quezada and Toledo, 2020). In lepidolite, it is predicted that the Li is bound between layers of AlO_6_ in an octahedral structure and SiO_4_ in a tetrahedral structure acting as predominantly charge supplementing ions. Therefore, the dominating bonding force is predicted to be highly ionic in these crystal structures (Su et al., 2019; Franco et al., 1973). As such, releasing Li from spodumene and lepidolite requires breaking high-energy aluminosilicate bonds. In jadarite, only lower energy Li–B bonds are required to be broken to release Li to the solution. Similarly, the bonding is described as low energy within the jadarite structure (Whitfield et al., 2007), indicating that it may be easier to release metals at ambient temperature in acidic conditions than the Li mineral types, as seen in both the bioleaching experiments and the uninoculated control (Ňancucheo et al., 2016). Abiotic leaching caused the decomposition of a significant proportion of jadarite to produce aqueous Li. This is further evidence of the lower stability of the mineral in acidic conditions due to lower activation pathways to bond-breaking reactions resulting in Li release.

### 3.6 Kinetic analysis

**Figure 7:**
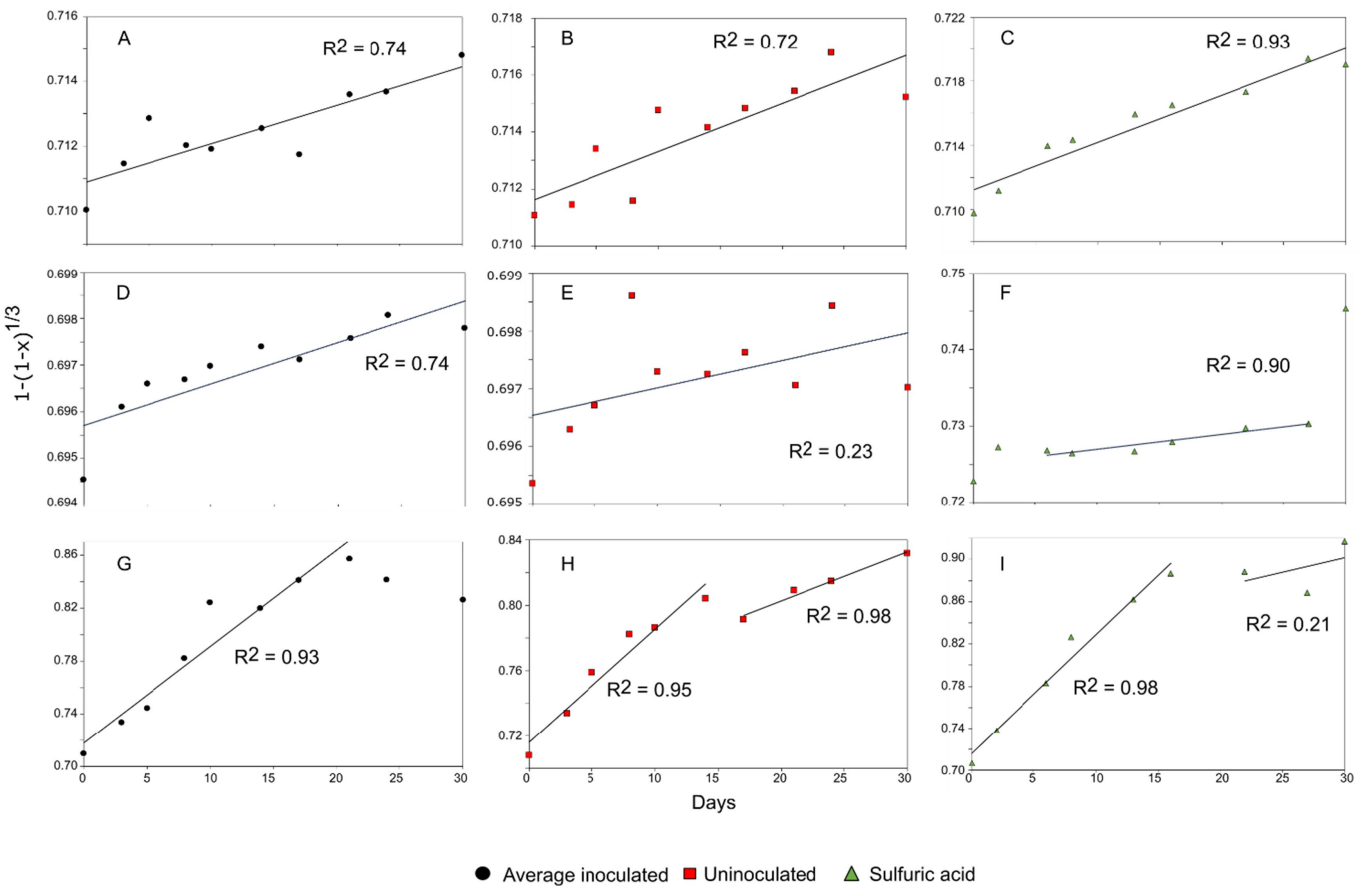
Linear regression of the chemical shrinking core model for the leaching of Li from lepidolite via (A) bioleaching R^2^ = 0.74, (B) uninoculated control R^2^ = 0.72 and (C) sulphuric acid leaching R^2^ = 0.93. Linear regression of the chemical shrinking core model for the leaching of Li from spodumene via (D) bioleaching R^2^ = 0.74, (E) uninoculated control R^2^ = 0.23, and (F) sulphuric acid leaching R^2^ = 0.90. Linear regression of the chemical shrinking core model for the leaching of Li from jadarite via (G) bioleaching R^2^ = 0.93, (H) uninoculated control R^2^ = 0.95, R^2^ = 0.98 and (I) sulphuric acid leaching R^2^ = 0.98, R^2^ = 0.21. The black circles represent bioleaching reactions, the red squares represent uninoculated controls, and the green triangles represent acid leaching.

The shrinking core kinetic model was used to model the reaction kinetics for each system. This model predicts whether acid leaching is the predominant mechanism of release for a given element but will not conform in the presence of catalysts such as biological mechanisms. This is useful for understanding the influence of bacteria in the bioleaching process compared to the acid leaching, as well as for understanding the extent of the reactions taking place. For both lepidolite and spodumene, the collected data does not adjust to the shrinking core model as denoted by the low R^2^ (R^2^ = 0.74), (Figures 7A,D). A multiphase reaction was difficult to predict, and the model further confirmed little to no reaction progression over 30 days, as indicated by shallow the gradients of the plotted results. The slopes of the plotted data were small for both lepidolite and spodumene (1 × 10^−4^ and 9 × 10^−5^, respectively), indicating low rate constants (gradient) and hence slow reaction times. The model showed good agreement for acid leaching of both minerals, with R^2^ values exceeding 0.9 in both cases, proving validity in the model for mineral–acid interactions. The contribution of the bacteria was probably very limited based on these values and helps to explain the lack of difference in aqueous Li concentration between the biotic experiments and the uninoculated controls.

Jadarite showed a poor fit to the chemically driven shrinking core model after the first 15 days of reaction, with no established rate constant and low correlation (R^2^ = 0.67) when plotted against the established model (Figure 7G). The contrasts in gradient between the two reaction phases indicated a much larger gradient and hence a greater reaction rate during the first stages of the model. There is a difference in gradient during the initial reaction stages and later days, with a more positive gradient in the linear regression in the second stage of the reaction. This implies that the quasi-steady state may have been reached from at least day 20 of the reaction, with a positive rate constant (gradient) and agreement with the model (Figures 7G–I). This is shown in the release of Li at a greater rate in the earlier stage of the reaction. The later reaction stages did not fit the model (R^2^ = 0.42), likely due to the contribution of the bacterial activity and formation of secondary products as well as the chemical leaching due to the nature of the kinetic model. There would be a poor fit when there is the contribution of the biological catalyst to the reaction rate. This is further emphasised by the elevated concentrations of Li recorded in the jadarite bioleaching from day 10 compared to the negative control (Figure 1) (Fogler, 2020). The model showed a good fit for the acid leaching of jadarite in the first reaction stages, which followed a similar trend to that of bioleaching. However, the Li concentration continued to increase slowly rather than plateauing as with the bioleaching system, indicated by the more positive gradient on the second stage of the acidic kinetic plot.

### 3.7 Scale-up of jadarite bioleaching

The feasibility of scale-up of the jadarite experiment was considered and deemed to have many barriers. The first is the volume of acid required to produce and maintain the pH 1–2 conditions needed for successful bioleaching. For this, a minimum of 0.9% of the total volume of the system needs to be 5.5 M H_2_SO_4_. A volume of 1,000 L would require 9 L of 5.5 M H_2_SO_4_ to initiate leaching and additional amounts throughout the process to neutralise components whose dissolution buffers the pH (e.g., the dolomite within the jadarite used in this study). This is less than the 1.3% required volume for acid leaching with H_2_SO_4_, which equates to 13 L per 1,000 L volume. While the Li release from bioleaching was lower than with H_2_SO_4_, the requirement for less acid would reduce the costs associated with the reaction. The rate of Li release by *At. ferrooxidans* was comparable to that of the acid leaching over 30 days. Typically, bioleaching can have slow reaction rates. For example, bioleaching of chalcopyrite (CuFeS_2_) by *At. ferrooxidans* can take up to 80 days to release 60–70% of available Cu (Zhao et al., 2013). Therefore, Li release is considerably fast through these experiments in comparison to other bioleaching processes. Another consideration is the formation of H_3_BO_3_, which poses additional hazards and its disposal would add cost and risk to the scale-up (likely to occur in all jadarite acid leaching experiments). Therefore, while Li release was enhanced by the presence of *At. ferrooxidans*, its scale-up would need careful consideration of environmental impact and waste management prior to use.

## 4 Conclusion

Bioleaching released up to 57% of Li from jadarite, but this required substantial additions of H_2_SO_4_ to maintain the pH below 2. The presence of the model Fe(II)/S-oxidising bacterium *A. ferroxidans* increased the rate of bioleaching of jadarite in early reaction stages, but the overall extent of Li leaching from jadarite, spodumene, and lepidolite was not significantly greater than abiotic acid leaching. The conformity of the spodumene and lepidolite data to the shrinking core model indicates a heterogenous, non-catalytically driven reaction where chemistry is the predominant driver. This supports previous findings that showed lepidolite to follow the same leaching trends as chemical leaching (Olaoluwa et al., 2023). The jadarite system did not conform to the chemical model for the kinetics in later reaction stages, indicating catalytic and biological contributions to the reaction. In conclusion, these results give preliminary evidence of the suitability of a (bio)leaching system for Li extraction from jadarite-containing minerals containing dolomite as a buffering contaminant and other competing metals in different mineral phases, with reaction times like that of chemical leaching methods. Bioleaching released 18% less Li than sulphuric acid leaching but showed increased selectivity with lower concentrations of B and Ca measured in solution. The lower concentration of B may be due to the formation of crystalline structures during the bioleaching. This could be beneficial when optimised to make further processing and concentrating of Li more effective. However, further studies on the extent of the formation of crystalline H_3_BO_3_ in the jadarite system should be considered prior to scale-up, as environmental implications and health effects of H_3_BO_3_ may be significant (Hadrup et al., 2021).

## Data Availability

The datasets presented in this study can be found in online repositories. The names of the repository/repositories and accession number(s) can be found at: Kirk, Rebecca (2024), “Bioleaching lithium from jadarite, spodumene and lepidolite using Acidiothiobacillus ferrooxidans”, Mendeley Data, V1, doi: 10.17632/djkf7tkm8d.1.
