# Supplementary material for: Bioleaching of lithium from jadarite, spodumene, and lepidolite using Acidiothiobacillus ferrooxidans
Source: Front Microbiol. 2024 Nov 13;15:1467408. doi: 10.3389/fmicb.2024.1467408 (PMC11622194; doi:10.3389/fmicb.2024.1467408)
Supplement: Supplementary file 1 [file Data_Sheet_1.DOCX]

Supplementary Material

Bioleaching lithium from jadarite, spodumene and lepidolite using *Acidiothiobacillus ferrooxidans*

Rebecca D. Kirk^1*^, Laura Newsome^1^, Carmen Falagan^1,2^, Karen A. Hudson-Edwards^1^

^1^Environment and Sustainability Institute and Camborne School of Mines, University of Exeter, Penryn Campus, Penryn, Cornwall, TR10 9FE, United Kingdom

^2^University of Portsmouth, Winston Churchill Avenue, Southsea, Portsmouth, PO1 2UP, United Kingdom.

*** Correspondence:**Rebecca Dawn Kirk

Rk519@exeter.ac.uk

# Supplementary Data

The datasets presented in this study can be found in online repositories. The name of the repository and accession numbers can be found below: Kirk, Rebecca (2024), “Bioleaching lithium from jadarite, spodumene and lepidolite using Acidiothiobacillus ferrooxidans”, Mendeley Data, V1, doi: 10.17632/djkf7tkm8d.1

# Supplementary Figures and Tables


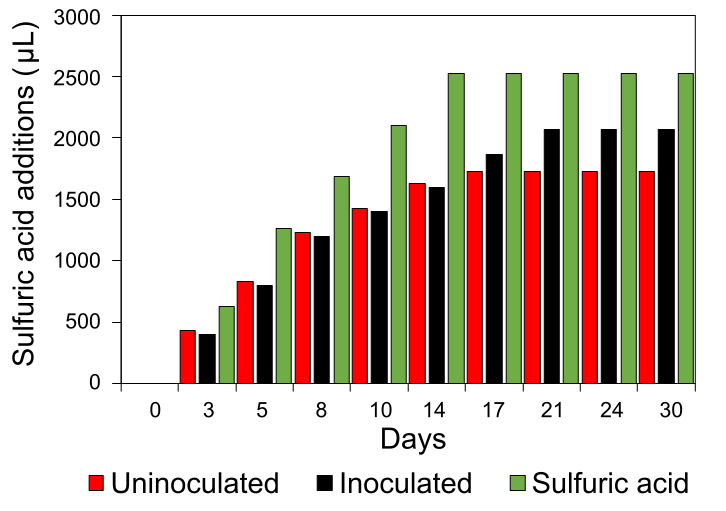


**Figure S1:** Cumulative additions of 5.5M H_2_SO_4_ to each jadarite leaching system over 30-day reaction time.

**Table S1:** Metal concentrations measured in jadarite, spodumene and lepidolite samples prepared via 4-acid digestion given to two significant figures. ‘<10’ indicates measurements below the levels of detection for the given element based on standards used for ICP-OES data analysis. ‘*’ measurement taken from 24 hour 70% HNO_3_ leach due to loss of B in open vessel 4-acid digestion of jadarite.

| **Concentration (µg/g)** | **Jadarite** | **Spodumene** | **Lepidolite** |
| --- | --- | --- | --- |
| **Al** | <10 | 10000 | 94000 |
| **B** | 59000* | <10 | <10 |
| **Ca** | 330 | <10 | <10 |
| **Fe** | 10 | <10 | 840 |
| **K** | 30 | 11 | 1100 |
| **Li** | 230 | 120 | 80 |
| **Mg** | 150 | <10 | <10 |
| **Mn** | <10 | <10 | <10 |
| **Na** | 27 | <10 | <10 |
| **S** | <10 | <10 | <10 |
| **P** | <10 | <10 | <10 |

**Table S2:** Concentrations of bioavailable Fe estimated through measurement of concentration leached from 0.5M HCl via the ferrozine assay on sediments for the Li bearing minerals measured in mM.

| **Mineral** | **Fe(II) (mM)** | **Fe (III) (mM)** | **Fe total (mM)** |
| --- | --- | --- | --- |
| Jadarite | 0.020 | 4.8 | 4.8 |
| Spodumene | 0.023 | 0.024 | 0.047 |
| Lepidolite | 0.031 | 0.023 | 0.054 |

**
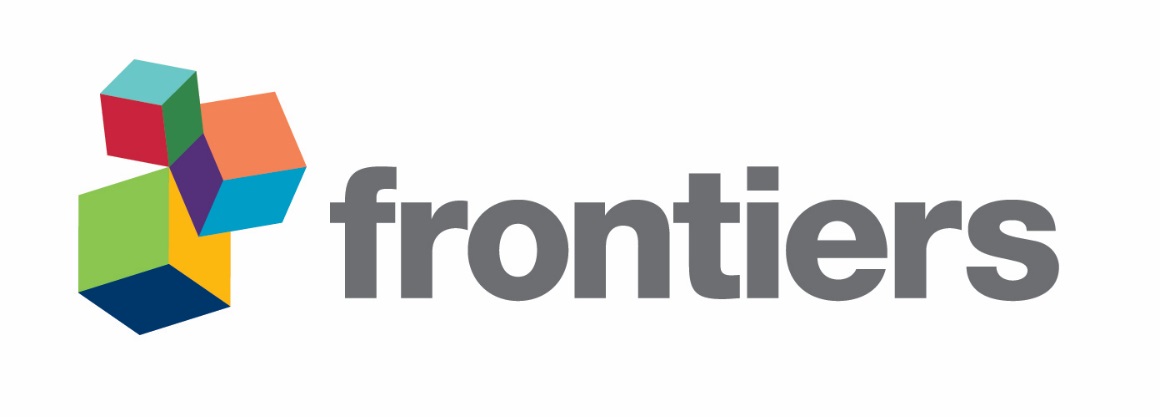
**
